# One-carbon metabolism in cancer immunity: T-cell fitness, epigenetic programming, and therapeutic opportunities

**DOI:** 10.3389/fimmu.2026.1867162

**Published:** 2026-07-13

**Authors:** Rongfei Wang, Naiwen Zhang, Runbing Xu, Zichen Xu, Hongbo Li

**Affiliations:** 1 Department of Breast and Thyroid Surgery, Jinhua People’s Hospital, Jinhua, Zhejiang, China; 2 Department of Hematology and Oncology, Dongzhimen Hospital, Beijing University of Chinese Medicine, Beijing, China; 3 Selwyn College, University of Cambridge, Cambridge, United Kingdom

**Keywords:** cancer immunity, epigenetic programming, immunometabolism, one-carbon metabolism, T-cell fitness

## Abstract

One-carbon metabolism has emerged as a critical interface between tumor metabolic adaptation and antitumor immunity. Beyond its canonical role in nucleotide biosynthesis and redox balance, this metabolic network regulates methyl-donor availability, epigenetic programming, and immune-cell state transitions within the tumor microenvironment. Recent studies show that tumor cells can outcompete T cells for methionine, thereby depleting intracellular S-adenosylmethionine, impairing histone methylation, and driving effector dysfunction or exhaustion. At the same time, tumor-intrinsic one-carbon enzymes such as MTHFD2 actively promote immune escape through PD-L1 upregulation and suppression of innate immune sensing. Serine-related pathways further shape the immune landscape in a context-dependent manner, supporting either immunosuppressive cell accumulation or enhanced tumor immunogenicity depending on the cellular compartment and metabolic state. Importantly, one-carbon metabolism also represents a therapeutic opportunity. Strategies including methionine restriction, formate supplementation, serine-pathway targeting, and methyl-donor modulation have shown potential to enhance antitumor immunity and improve responses to immune checkpoint blockade. However, because one-carbon metabolism is required by both tumor cells and immune cells, effective intervention will require careful attention to cell-type specificity, metabolic context, and therapeutic timing. In this review, we discuss how one-carbon metabolism governs T-cell fitness, epigenetic regulation, and the broader tumor immune ecosystem, and we highlight emerging translational strategies for exploiting this pathway in cancer immunotherapy.

## Highlights

- One-carbon metabolism shapes antitumor immunity by regulating nutrient competition, methyl-donor availability, and T-cell functional state.
- Tumor-intrinsic one-carbon pathways promote immune escape through checkpoint regulation and suppression of innate immune sensing.
- Therapeutic modulation of methionine, serine, formate, and methyl-donor metabolism may improve immunotherapy efficacy but requires context-specific design.

## 1 Introduction

Antitumor immunity is commonly interpreted through the lens of immune checkpoints, cytokine networks, and the abundance or spatial distribution of immune-cell subsets (1–5). Although these frameworks remain essential, they are insufficient to explain why tumor-infiltrating lymphocytes often become dysfunctional even in tumors that appear immunologically “inflamed.” Increasing evidence indicates that immune failure in cancer is also a metabolic problem (6–8). Within the tumor microenvironment, malignant cells and immune cells coexist under conditions of nutrient limitation, oxidative stress, and metabolite accumulation. Under these conditions, the availability of specific nutrients is not a passive background variable but an active determinant of immune-cell state, lineage stability, and effector competence.

Among the metabolic circuits implicated in cancer immunity, one-carbon metabolism is emerging as particularly important because it sits at the intersection of biosynthesis, redox control, and epigenetic regulation (9–13). In tumor cells, one-carbon metabolism supports proliferation, survival, and adaptation to environmental stress. In T cells, the same pathway helps sustain activation-induced anabolic demand, nucleotide production, methyl-donor availability, and inflammatory output (14–17). This dual relevance makes one-carbon metabolism uniquely suited to shape both sides of the tumor–immune interaction. Rather than acting solely as a housekeeping pathway, it can influence whether the tumor microenvironment favors immune activation or immune escape. This concept is illustrated by several key studies. Bian and colleagues showed that tumor cells can deplete methionine through high expression of the transporter SLC43A2, thereby reducing intracellular methionine and S-adenosylmethionine in CD8+ T cells and impairing histone methylation and effector function. Sugiura and colleagues demonstrated that the one-carbon enzyme MTHFD2 acts as a metabolic checkpoint in activated T cells, integrating one-carbon flux with purine metabolism and inflammatory signaling. Shang and colleagues further extended the field by showing that tumor-intrinsic MTHFD2 activity can promote immune evasion through PD-L1 upregulation. Together, these studies support a broader view in which one-carbon metabolism is not merely a biosynthetic pathway, but an immunoregulatory system linking nutrient availability, epigenetic programming, and antitumor immunity.

One-carbon metabolism refers to an interconnected network centered on the transfer and utilization of single-carbon units for anabolic and regulatory purposes. In mammalian cells, this network includes serine–glycine metabolism, the folate cycle, the methionine cycle, S-adenosylmethionine-dependent methylation reactions, and formate as a transferable one-carbon donor (18–22). These branches are functionally coupled rather than isolated: serine provides one-carbon units to the folate cycle, folate-mediated reactions support nucleotide biosynthesis and redox balance, and the methionine cycle generates methyl donors that influence chromatin state and gene expression (23–26). From an immunological perspective, the importance of this network lies in its ability to support the rapid metabolic remodeling required for lymphocyte activation. Activated T cells must expand biomass, synthesize nucleotides, and sustain transcriptional programs that underlie proliferation and cytokine production. One-carbon metabolism contributes directly to these processes by providing substrates for *de novo* purine synthesis and by maintaining methyl-donor pools required for DNA and histone methylation (27–29). Because T-cell activation is tightly coupled to transcriptional and epigenetic remodeling, disturbances in one-carbon flux can translate quickly into impaired cell-state transitions or loss of effector potential. Beyond proliferation, one-carbon metabolism also shapes immune-cell identity, metabolic resilience, and tumor–immune competition. Rather than treating individual studies as isolated observations, this review organizes the field around three connected layers: nutrient competition between tumor cells and immune cells, methyl-donor-dependent epigenetic programming, and context-specific therapeutic modulation. This structure allows the same metabolic pathway to be interpreted across immune-cell-intrinsic, tumor-cell-intrinsic, and ecosystem-level mechanisms.

This review does not aim to provide a general overview of one-carbon metabolism in cancer biology. Instead, it focuses specifically on how one-carbon metabolism shapes cancer immunity through three interconnected themes: T-cell fitness, epigenetic programming, and therapeutic opportunity. We first examine how one-carbon flux supports activated T cells and regulates lineage-specific immune states. We then discuss how nutrient competition and tumor-intrinsic one-carbon programs drive immune dysfunction and immune escape. Finally, we evaluate the translational potential of targeting or supporting one-carbon metabolism to improve antitumor immunity and immunotherapy response. Particular attention is given to five issues that increasingly define the field: nutrient competition between tumor cells and immune cells; tumor-intrinsic metabolic programs that regulate checkpoint expression and immune sensing; one-carbon-dependent control of T-cell fate and function; the extension of these effects to the broader tumor immune ecosystem, including Tregs and myeloid cells; and the emerging concept of metabolic intervention as an adjunct to immunotherapy. By organizing the discussion around these themes, this review seeks to position one-carbon metabolism as a unifying framework for understanding how metabolism and immunity co-determine cancer progression and therapeutic response. **Table 1** summarizes the major branches of one-carbon metabolism that connect nutrient availability to T-cell fitness, epigenetic programming, and tumor immune escape.

**Table 1:** Major one-carbon metabolic nodes linking tumor metabolism to cancer immunity.

Metabolic branch/node	Representative components	Major immunological function	Cancer-immunity relevance	Key references
Methionine cycle	Methionine, SAM, SAH	Supplies methyl groups for DNA/histone/protein methylation	Controls T-cell effector programming, exhaustion-associated transcription, and innate sensing thresholds	Bian 2020 (51); Pandit 2023 (63); Fang 2023 (115)
Serine–glycine metabolism	Serine, glycine, SHMT	Provides one-carbon units for biosynthesis and redox coupling	Supports immune-cell state control; may favor Treg accumulation in serine-rich tumors	Kurniawan 2020 (40); Ma 2024 (79)
Folate cycle	MTHFD2, folate intermediates	Couples one-carbon transfer to nucleotide synthesis and metabolic signaling	Regulates T-cell fate and can promote tumor immune escape via PD-L1	Sugiura 2022 (34); Shang 2021 (66)
Formate metabolism	Formate as transferable 1C donor	Supports one-carbon sufficiency under metabolic stress	Enhances CD8+ T-cell fitness and improves PD-1 blockade response	Rowe 2023 (45); Phelps 2025 (46)
*De novo* serine synthesis	PHGDH, PSPH	Sustains serine supply and downstream one-carbon flux	Can support immune-cold tumor states; targeting may enhance ICB efficacy	Peng 2023 (58); Wang 2025 (111)
Methylation-dependent immune regulation	Histone methylation, DNA methylation, cGAS methylation	Translates nutrient state into chromatin and immune-sensing programs	Links nutrient competition to T-cell dysfunction and tumor immune visibility	Bian 2020 (51); Fang 2023 (115)

## 2 One-carbon metabolism as a determinant of t-cell fitness and immune cell fate

### 2.1 MTHFD2 as a metabolic checkpoint in T cells

Activation of T cells triggers a profound metabolic transition in which quiescent lymphocytes shift toward a highly anabolic state (30–33). This transition requires increased flux through pathways that support nucleotide biosynthesis, mitochondrial adaptation, and inflammatory signaling. One-carbon metabolism is well positioned to meet these demands because it links serine catabolism and folate-mediated one-carbon transfer to purine synthesis and methyl-group generation. Within this network, MTHFD2 has emerged as a particularly important enzyme in activated T cells. Rather than functioning as a passive metabolic component, MTHFD2 appears to operate as a regulatory checkpoint that coordinates metabolic input with effector cell behavior.

Sugiura and colleagues demonstrated that MTHFD2 is induced in activated T cells and regulates *de novo* purine synthesis as well as signaling pathways associated with proliferation and inflammatory cytokine production. Importantly, this effect is not limited to generic biomass expansion (34). Their work showed that MTHFD2 influences the balance between effector and regulatory T-cell states, indicating that one-carbon metabolism participates directly in lineage specification and functional polarization. This finding significantly expands the conceptual role of one-carbon metabolism in immunity: it is not merely permissive for T-cell activation, but instructive in shaping how activated T cells behave. Together, these findings suggest that one-carbon metabolism contributes to lineage-specific immune fitness through distinct mechanisms in different T-cell subsets. In effector T cells, it supports expansion and inflammatory competence; in regulatory T cells, it helps preserve a stable suppressive program. Therefore, one-carbon metabolism should be viewed not simply as a growth pathway for activated lymphocytes, but as a central determinant of T-cell fate and immune-cell specialization.

### 2.2 Serine metabolism and the preservation of regulatory T-cell identity

Regulatory T cells are often discussed as a relatively stable suppressive population, but this stability is metabolically maintained rather than intrinsically guaranteed. Their persistence depends on a carefully balanced metabolic program that avoids excessive inflammatory activation while preserving FoxP3 expression and suppressive function (35–38). Serine metabolism is part of this balancing system. Because serine contributes to both glutathione synthesis and one-carbon metabolism, changes in its intracellular handling can alter redox homeostasis, anabolic flux, and transcriptional stability (39). Kurniawan and colleagues provided an important mechanistic framework for this concept by showing that glutathione restricts serine metabolism in Tregs (40). In the absence of appropriate glutathione-mediated control, Tregs displayed increased serine metabolism, aberrant mTOR activation, enhanced proliferation, and reduced FoxP3 expression. Notably, limiting serine availability restored suppressive capacity in this context, indicating that Treg identity depends not on maximal nutrient utilization but on regulated metabolic restraint. This finding is highly relevant to cancer immunity because it shows that one-carbon-linked nutrients can tune immune-cell state in a selective and lineage-dependent manner. This section therefore emphasizes the immune-cell-intrinsic requirement for controlled serine utilization in maintaining Treg identity. The broader question of how serine-rich tumor niches shape Treg accumulation at the ecosystem level is discussed separately below.

### 2.3 Formate availability and CD8+ T-cell metabolic resilience

One-carbon units should not be viewed only as intracellular intermediates generated from serine. They can also function as limiting environmental inputs that shape T-cell performance in tumors. This is particularly relevant for CD8+ T cells, which must sustain proliferation, cytotoxicity, and cytokine production under conditions of persistent antigen exposure and nutrient deprivation (41–44). Even when checkpoint blockade partially reinvigorates exhausted T cells, their response may remain constrained by insufficient metabolic support. In this setting, one-carbon insufficiency may represent an underappreciated barrier to full functional recovery. Rowe and colleagues directly addressed this possibility by showing that one-carbon metabolism is enhanced in antigen-stimulated T cells and that therapeutic formate supplementation can improve CD8+ T-cell fitness in tumors (45). In murine tumor models, formate supplementation increased the antitumor efficacy of PD-1 blockade, supporting the idea that exogenous one-carbon support can help reinvigorated T cells overcome a metabolic bottleneck within the tumor microenvironment. Conceptually, this study is important because it shifts the therapeutic conversation away from tumor-directed metabolic inhibition alone and toward the possibility of selectively supporting immune-cell metabolism.

**Figure 1:**
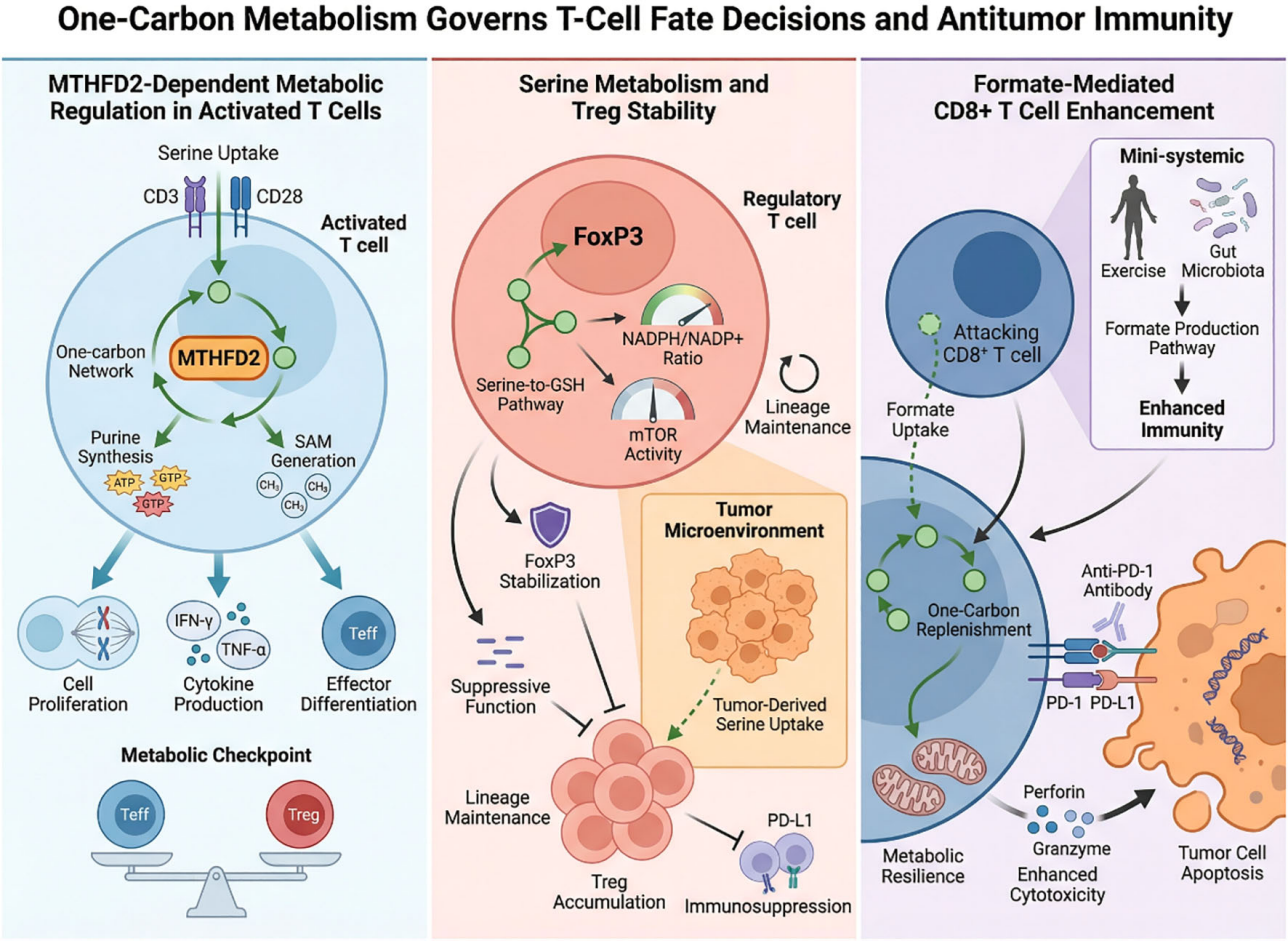
One-carbon metabolism as a determinant of T-cell fitness and immune cell fate. One-carbon metabolism supports activated T-cell proliferation, purine synthesis, and inflammatory function through MTHFD2-dependent flux and methyl-donor generation. In regulatory T cells, controlled serine metabolism preserves FoxP3 stability and suppressive identity, whereas serine-rich tumors favor Treg accumulation. Formate supplementation, including microbiota-derived formate, enhances CD8+ T-cell metabolic resilience and improves antitumor immunity, including responses to PD-1 blockade.

Recent work further broadens this idea by linking host physiology and the microbiota to one-carbon availability. Phelps and colleagues reported that exercise can stimulate microbial one-carbon metabolism and increase formate production, which in turn enhances CD8+ T-cell antitumor immunity and improves immunotherapy efficacy (46). Although this finding extends beyond tumor-local metabolism, it underscores an important principle for the field: one-carbon metabolism is not only a cell-intrinsic pathway but also a systemic immunometabolic axis influenced by host behavior and microbiota-derived metabolites. This perspective opens the door to new strategies in which metabolic resilience of antitumor T cells is improved through dietary, microbial, or lifestyle-associated modulation of one-carbon inputs. **Figure 1** illustrates how one-carbon metabolism regulates T-cell fate, functional fitness, and immune specialization within the tumor microenvironment. It also highlights that one-carbon inputs can act as therapeutic leverage points to enhance CD8+ T-cell-mediated antitumor immunity.

## 3 Nutrient competition in the tumor microenvironment: methionine restriction as a driver of T-cell dysfunction

### 3.1 Tumor cells outcompete T cells for methionine

The tumor microenvironment is increasingly recognized as a site of metabolic competition in which malignant cells and immune cells contend for limited nutrients. Among these nutrients, methionine occupies a particularly important position because it is not only required for protein synthesis but also serves as the precursor for S-adenosylmethionine (SAM), the principal methyl donor used in DNA, RNA, and histone methylation reactions (47–50). As a result, methionine availability directly influences not only cell growth but also the epigenetic machinery that sustains T-cell activation and effector function. In the context of cancer, this makes methionine a strategic metabolic resource whose depletion can disable antitumor immunity at multiple levels.

A landmark study by Bian and colleagues provided compelling evidence that tumor cells can outcompete CD8+ T cells for methionine through high expression of the transporter SLC43A2 (51). In this model, malignant cells preferentially scavenge extracellular methionine, thereby lowering intracellular methionine and SAM levels in neighboring T cells. The result is a marked reduction in histone methylation in CD8+ T cells, accompanied by impaired transcriptional support for effector programs and reduced IFN-γ production (52–55). This work established a clear mechanistic chain linking tumor nutrient acquisition to immune dysfunction: enhanced methionine uptake by cancer cells deprives T cells of a critical one-carbon substrate, disrupts methyl-donor homeostasis, and ultimately compromises antitumor activity. This finding has broad conceptual implications. First, it argues that immune failure in tumors cannot be understood solely in terms of inhibitory receptors or suppressive cytokines; it must also be interpreted as a consequence of unequal access to metabolites. Second, it reveals that one-carbon metabolism is not merely a tumor-intrinsic biosynthetic program but a shared ecological resource over which tumor cells and immune cells compete. Third, it suggests that the outcome of this competition is particularly consequential for T cells because methionine deprivation affects not only energy status or protein synthesis but also the epigenetic infrastructure required for sustained effector identity. In this sense, methionine competition represents a highly efficient strategy of immune evasion: by monopolizing a single nutrient, tumor cells can simultaneously weaken T-cell function, destabilize transcriptional programs, and blunt antitumor immunity.

From a broader perspective, the SLC43A2 story reframes nutrient competition as an immunoregulatory mechanism rather than a passive by-product of tumor growth (56–58). Tumor cells do not merely consume methionine because they proliferate rapidly; they can use methionine acquisition to shape the functional state of infiltrating T cells. This idea is especially important for one-carbon metabolism because methyl-donor availability sits at the interface of metabolism and gene regulation. Thus, in the methionine-poor tumor microenvironment, T-cell dysfunction is not simply a matter of “starvation,” but a failure to maintain the epigenetic and transcriptional architecture required for durable antitumor responses.

### 3.2 Methionine metabolism and T-cell exhaustion in cancer

The consequences of methionine deprivation extend beyond a simple reduction in effector output. An emerging body of evidence suggests that persistent limitation of methionine in tumors can actively bias T cells toward dysfunctional and exhausted states (59–61). This distinction is important. Reduced cytokine production or proliferative fitness might be reversible signs of metabolic insufficiency, whereas exhaustion reflects a deeper cell-state transition characterized by sustained inhibitory receptor expression, altered chromatin accessibility, and progressive loss of effector competence. Methionine metabolism appears to participate in this transition by controlling the epigenetic resources needed to resist dysfunctional programming.

Hung and colleagues provided a major contribution to this concept in hepatocellular carcinoma, where they showed that tumor methionine metabolism is associated with T-cell exhaustion and clinical outcome (62). Their study linked methionine-related metabolic rewiring in HCC to an exhausted T-cell state across patient cohorts, supporting the idea that methionine metabolism is not simply a biochemical feature of tumor cells but a determinant of immune dysfunction in human disease. Importantly, this work moved the field beyond the notion that methionine competition merely attenuates T-cell activity; it suggested instead that tumor methionine metabolism helps establish a durable exhaustion program in the tumor microenvironment. This concept was extended further by Pandit and colleagues, who showed that methionine consumption by cancer cells can drive a progressive exhausted phenotype in CD4+ T cells in hepatocellular carcinoma. Their study demonstrated that methionine deprivation in CD4+ T cells reduces H3K79 dimethylation, downregulates AMPK, and increases PD-1 expression, collectively impairing antitumor immunity (63). Notably, this work broadened the relevance of methionine competition beyond CD8+ cytotoxic lymphocytes and established that CD4+ T-cell dysfunction can also be metabolically imprinted by the tumor. This is mechanistically important because CD4+ T cells are essential for coordinating antitumor immunity, supporting CD8+ T-cell responses, and shaping the broader immune landscape.

Together, these studies support a more forceful interpretation of methionine deprivation in cancer: it does not simply weaken T-cell function in a quantitative sense, but can qualitatively redirect T cells toward dysfunctional and exhausted states. This progression likely reflects the chronic nature of nutrient stress in tumors. Under sustained methionine limitation, the loss of methyl-donor availability is expected to gradually reshape transcriptional and epigenetic programs, favoring inhibitory receptor expression and diminishing the ability of T cells to maintain an effector identity. Thus, methionine metabolism serves as a bridge between metabolic competition and immune-state remodeling. This section also carries an important translational message. If methionine competition contributes to exhaustion programming, then restoring methionine access or preventing tumor-specific methionine sequestration may do more than transiently improve T-cell metabolism; it may help prevent or reverse the establishment of dysfunctional cell states. In that sense, methionine metabolism represents not only a metabolic axis of immune suppression but also a potential point of intervention for rescuing antitumor T-cell competence.

### 3.3 Epigenetic consequences of methionine restriction in T cells

One of the most important reasons methionine competition has such profound immunological effects is that methionine is coupled directly to epigenetic regulation through the methionine cycle and SAM production. In T cells, SAM is required to support methyltransferase reactions that maintain DNA and histone methylation patterns associated with activation, cytokine expression, and lineage stability (64, 65). When methionine availability falls, the resulting drop in SAM can rapidly impair this epigenetic support system. This makes one-carbon metabolism a particularly powerful conduit through which extracellular nutrient conditions are translated into intracellular changes in cell fate and gene regulation. Bian and colleagues showed that methionine restriction caused by tumor SLC43A2 expression lowers the methyl-donor pool in CD8+ T cells and reduces histone methylation, with downstream impairment of effector gene expression and IFN-γ production. This finding was significant not only because it explained why methionine competition suppresses T-cell function, but also because it placed histone methylation at the center of the mechanism. The key insight is that nutrient competition is not merely metabolic in a narrow sense; it is epigenetic in consequence. In T cells, reduced access to methionine means reduced access to the methyl marks needed to preserve a functional effector program.

**Figure 2:**
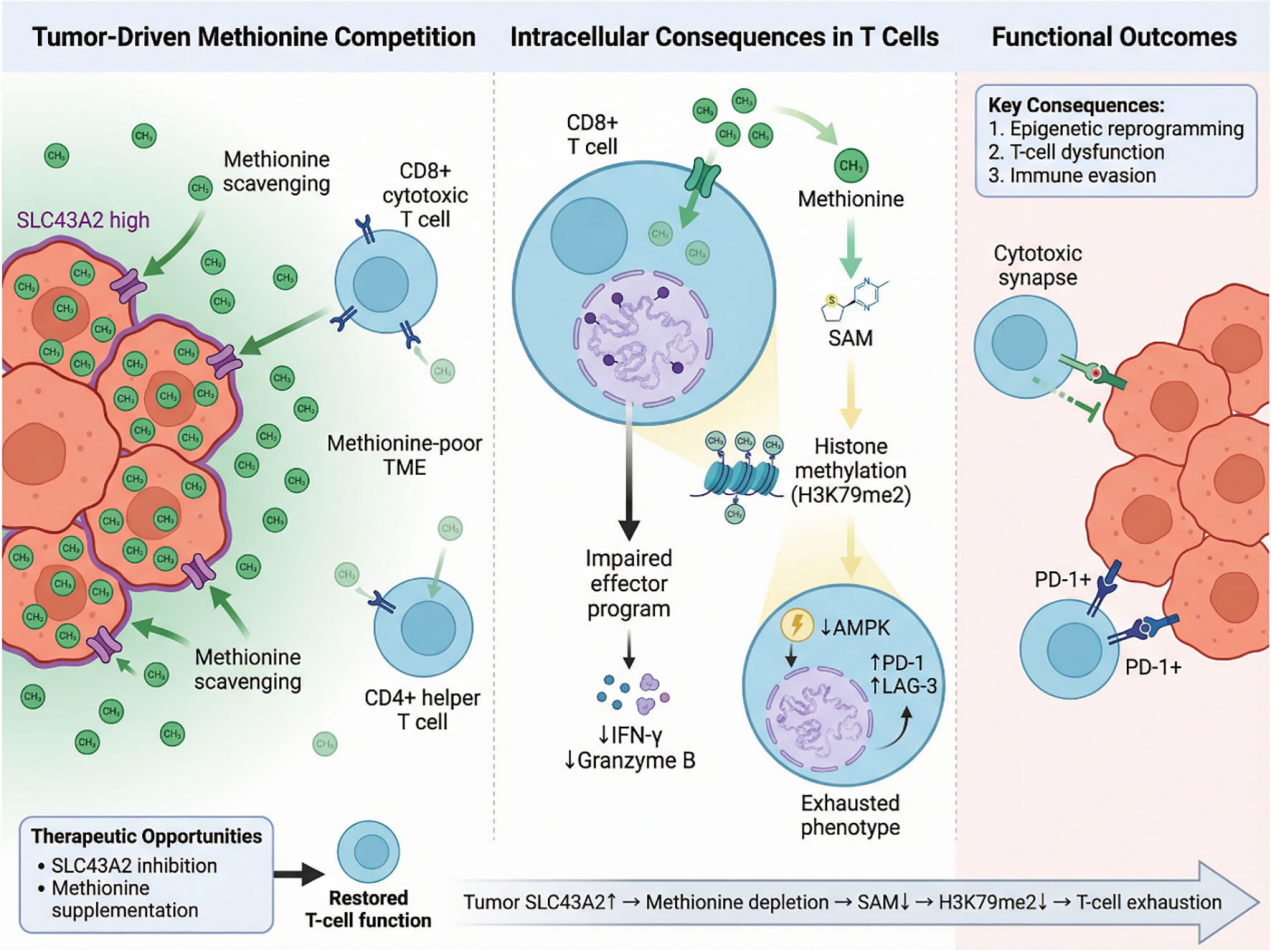
Tumor methionine competition drives T-cell dysfunction and exhaustion. Tumor cells preferentially consume methionine through SLC43A2, creating a methionine-poor microenvironment for infiltrating T cells. Reduced methionine availability lowers intracellular SAM and histone methylation, impairing CD8+ T-cell effector function and promoting exhaustion-associated programs in CD4+ T cells. These epigenetic and functional changes weaken antitumor immunity and facilitate tumor immune evasion.

Pandit and colleagues reinforced this principle in CD4+ T cells by showing that methionine deprivation lowers H3K79me2, decreases AMPK expression, and promotes PD-1 upregulation. This study is especially useful conceptually because it provides a concrete example of how a specific histone mark can link nutrient deficiency to an exhaustion-associated transcriptional phenotype. The implication is that chronic methionine shortage can epigenetically bias T cells away from metabolic resilience and toward inhibitory receptor expression. Such findings elevate methionine metabolism from a simple nutrient-supply issue to a key regulator of immune cell-state stability. These findings indicate that methionine deprivation should be interpreted as an epigenetic stress signal rather than simple nutrient scarcity. By altering methyl-donor availability and chromatin regulation, methionine restriction may convert transient metabolic stress into durable T-cell dysfunction. **Figure 2** illustrates how tumor-driven methionine sequestration acts as an immunoregulatory mechanism rather than a passive metabolic by-product. By reducing methyl-donor availability in T cells, methionine competition epigenetically reprograms infiltrating lymphocytes toward dysfunction and exhaustion.

## 4 Tumor-intrinsic one-carbon metabolism actively drives immune escape

### 4.1 Folate cycle enzymes as immune escape regulators

Although nutrient competition provides one powerful explanation for how one-carbon metabolism suppresses antitumor immunity, it is not the whole story. Tumor cells do not merely restrict immune responses indirectly by depriving T cells of metabolites; they can also use their own one-carbon metabolic machinery to actively construct immune-evasive phenotypes. This distinction is important because it expands the field from an ecological model of nutrient competition to a tumor-intrinsic model of immunoregulation. In this framework, one-carbon enzymes are not only biosynthetic components that support cell proliferation, but also regulators of checkpoint signaling and immune communication.

A pivotal study by Shang and colleagues exemplifies this concept. They showed that the folate cycle enzyme MTHFD2 promotes cancer immune evasion through PD-L1 upregulation (66). This finding directly links tumor one-carbon metabolism to immune checkpoint control and establishes that a metabolic enzyme can act upstream of a canonical immune-suppressive pathway. The significance of this result goes beyond the specific MTHFD2–PD-L1 axis. It demonstrates that tumor one-carbon metabolism is not merely growth-supportive, but checkpoint-regulatory. In other words, tumor cells can use one-carbon flux not only to meet anabolic demand but also to modify how they are perceived and attacked by the immune system. This observation has several implications for the field. First, it shows that one-carbon metabolism can influence immune escape even in the absence of direct nutrient competition with T cells (67–70). Second, it suggests that metabolic enzymes traditionally viewed through the lens of proliferation may also function as upstream nodes in immunoregulatory signaling networks. Third, it raises the possibility that targeting tumor one-carbon metabolism could simultaneously impair tumor growth and reduce immune suppression. However, as later sections will discuss, such strategies must be approached carefully because one-carbon pathways are also required by immune cells themselves. Conceptually, the MTHFD2 study helps define a broader principle: tumor metabolism can be immunologically instructive. Rather than being a passive reflection of tumor growth status, the metabolic state of the cancer cell can directly shape checkpoint expression, immune sensing, and susceptibility to immune attack. This makes tumor-intrinsic one-carbon metabolism a central component of the immune landscape of cancer rather than a merely intracellular metabolic feature.

### 4.2 *De novo* serine synthesis and immune responsiveness

Another important branch of tumor-intrinsic one-carbon regulation involves *de novo* serine synthesis. Serine is a critical donor to the one-carbon network, and its biosynthesis supports nucleotide production, redox control, and anabolic adaptation. In cancer, upregulation of serine synthesis is often discussed as a mechanism of growth support, but recent studies indicate that it also has direct consequences for immune responsiveness (71, 72). This is particularly relevant in tumors that rely on *de novo* serine synthesis to maintain metabolic plasticity under nutrient-limited conditions.

Peng and colleagues showed that downregulation of phosphoserine phosphatase (PSPH), a key enzyme in the serine synthesis pathway, potentiates tumor immune environments and enhances response to immune checkpoint blockade (73). This finding suggests that active serine synthesis contributes to an immune-suppressive or immune-cold tumor state, and that interrupting this pathway can remodel the tumor microenvironment in favor of antitumor immunity. The implication is that serine biosynthesis is not simply a metabolic adaptation for tumor survival; it is also part of the machinery through which tumors reduce immune vulnerability. Saha and colleagues provided a complementary perspective by showing that serine depletion in cancer cells activates cGAS–STING signaling and promotes antitumor immunity (74). Their study indicates that restricting serine availability can increase tumor immunogenicity and improve responsiveness to immune checkpoint inhibitors. Mechanistically, this reinforces the idea that serine metabolism influences more than biomass production: it affects innate immune sensing within tumor cells themselves. As a result, tumor serine metabolism can help determine whether cancer cells remain immunologically silent or become more visible to the immune system.

Taken together, these studies position *de novo* serine synthesis as a regulator of immune responsiveness in tumors. When active, serine biosynthesis may support an immune-refractory state by sustaining anabolic and signaling programs that buffer tumors against immune attack. When disrupted, the same pathway may expose latent immunogenicity and enhance the efficacy of immunotherapy. This makes serine synthesis enzymes attractive translational targets, particularly in combination with checkpoint blockade. At the same time, these findings caution against overly simplistic interpretations of serine metabolism, because its immune effects appear to depend strongly on the cellular compartment and metabolic context in which it is perturbed.

### 4.3 Serine abundance versus serine deprivation: a context-dependent axis of tumor immunogenicity

One of the most interesting features of serine metabolism in cancer immunity is its bidirectional and context-dependent behavior (75–78). On the one hand, serine-rich tumors may create conditions that preferentially support immunosuppressive populations. On the other hand, serine deprivation in tumor cells may promote innate immune activation and improve immunotherapy response. These seemingly opposite effects are not necessarily contradictory. Rather, they suggest that the immunological consequences of serine metabolism depend on which cell type experiences the metabolic change, how severe the perturbation is, and which signaling pathways are engaged downstream.

**Figure 3:**
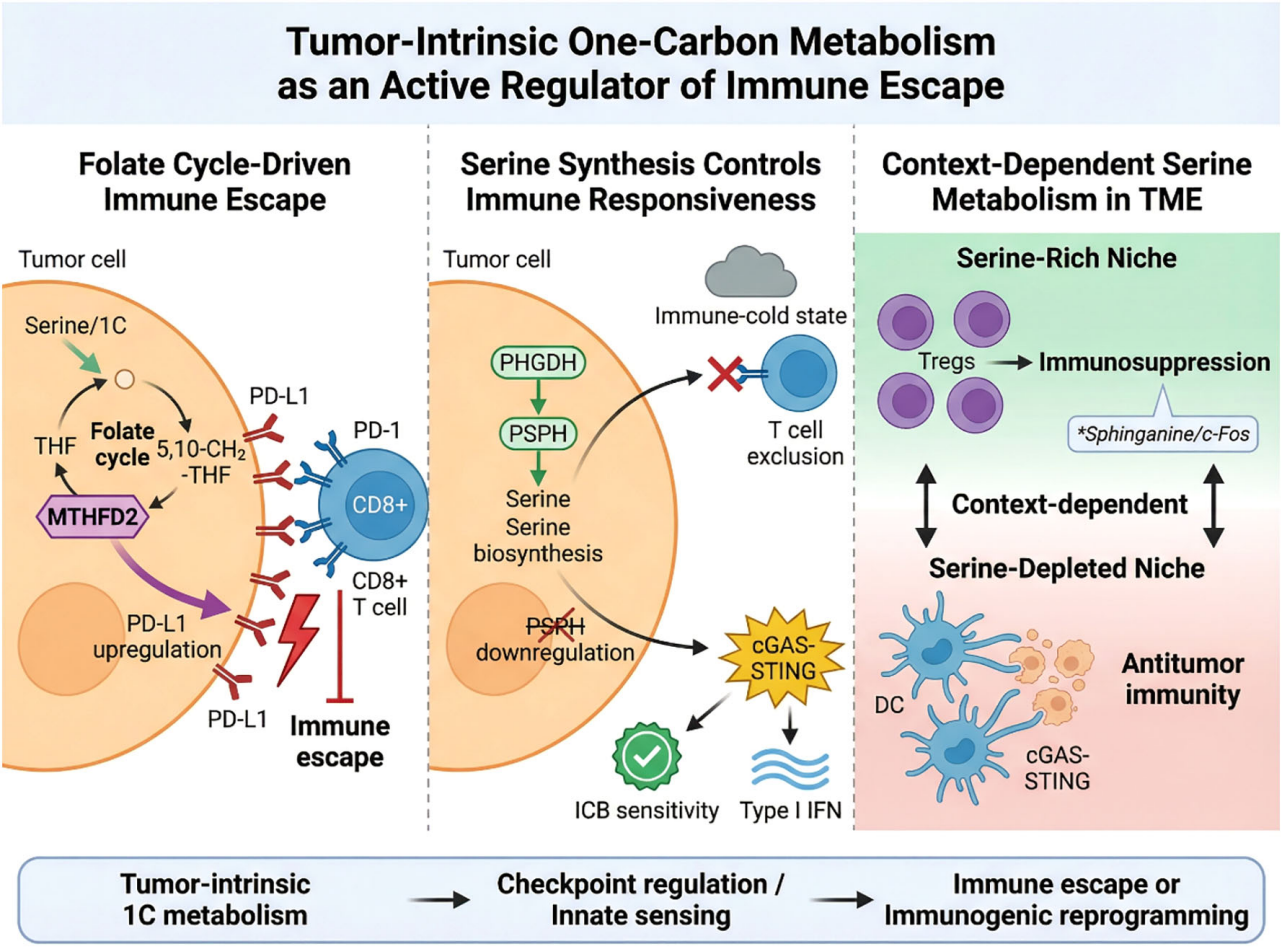
Tumor-intrinsic one-carbon metabolism actively regulates immune escape. Folate-cycle activity, particularly MTHFD2, promotes PD-L1 upregulation and checkpoint-mediated immune evasion. *De novo* serine synthesis supports immune-refractory tumor states, whereas serine depletion can activate cGAS–STING signaling and increase tumor immunogenicity. In parallel, serine-rich tumor niches may favor Treg accumulation, highlighting the bidirectional and context-dependent immunological effects of tumor-intrinsic one-carbon metabolism.

Ma and colleagues showed that serine enrichment in tumors promotes regulatory T-cell accumulation through sphinganine-mediated regulation of c-Fos (79). This study is especially notable because it demonstrates that the impact of serine extends beyond classical one-carbon biochemistry. Tumor serine abundance can alter the metabolic environment in a way that favors the accumulation of suppressive immune cells, thereby reinforcing immune escape at the ecosystem level. In this setting, serine availability functions as a selective force that shapes the cellular composition of the tumor microenvironment. In contrast, Saha and colleagues found that serine depletion in cancer cells activates cGAS–STING signaling and enhances antitumor immunity. Here, lowering serine does not primarily act on immune cells directly; instead, it appears to destabilize tumor metabolic homeostasis in a manner that increases immunogenic signaling. This distinction is crucial. Serine enrichment and serine deprivation can generate different immune outcomes because they operate through different cellular targets and mechanistic layers: one through ecosystem support of immunosuppressive cells, the other through tumor-cell intrinsic activation of innate immune sensing (80–83). This context dependence has important implications for both theory and therapy. It suggests that serine metabolism cannot be classified simply as either immunosuppressive or immunostimulatory. Instead, it should be understood as a tunable axis whose effects depend on cell type, nutrient level, pathway branching, and signaling context. For the purposes of cancer immunology, this means that serine metabolism may simultaneously contribute to tumor immune escape in one compartment while creating therapeutic vulnerabilities in another. Future work will need to define these contexts more precisely in order to exploit serine-directed interventions without inadvertently supporting suppressive immune programs. **Table 2** highlights representative original studies showing that one-carbon metabolism regulates cancer immunity through nutrient competition, T-cell fate control, tumor-intrinsic immune escape, and therapeutic modulation. **Figure 3** illustrates that tumor-intrinsic one-carbon metabolism is not merely anabolic but actively shapes immune escape through checkpoint regulation and innate immune sensing. It also highlights the context-dependent effects of serine metabolism, which can either support immunosuppressive tumor ecosystems or expose tumor immunogenic vulnerabilities.

**Table 2:** Representative original studies defining the role of one-carbon metabolism in cancer immunity.

Study	Model/cancer context	Main finding	Immunological implication	Suggested section
Bian et al. (51)	Multiple tumor models	Tumor SLC43A2 outcompetes CD8+ T cells for methionine, lowering intracellular methionine and methyl-donor availability and impairing histone methylation	Nutrient competition directly suppresses T-cell effector function	Methionine competition/T-cell dysfunction
Kurniawan et al. (40)	Treg biology	Glutathione restricts serine metabolism to preserve Treg function	Serine/redox coupling stabilizes suppressive T-cell identity	T-cell fate/Treg metabolism
Hung et al. (62)	Hepatocellular carcinoma	Tumor methionine metabolism is linked to exhausted T-cell states in HCC	Methionine metabolism contributes to durable immune dysfunction	T-cell exhaustion
Shang et al. (66)	Tumor-intrinsic model	MTHFD2 promotes immune evasion through PD-L1 upregulation	Folate-cycle enzymes can regulate checkpoint biology	Tumor-intrinsic immune escape
Sugiura et al. (34)	Activated T cells	MTHFD2 is a metabolic checkpoint controlling effector and regulatory T-cell fate and function	One-carbon flux shapes lineage-specific immune behavior	T-cell fitness/metabolic checkpoint
Pandit et al. (63)	Hepatocellular carcinoma	Tumor methionine consumption drives a CD4+ T-cell exhausted phenotype with epigenetic changes	Methionine deprivation promotes exhaustion-like programming beyond CD8+ T cells	Methionine restriction and epigenetic exhaustion
Fang et al. (115)	Tumor/innate sensing model	Methionine restriction activates cGAS by limiting methylation-dependent repression	One-carbon targeting can enhance innate immune activation	Therapeutic opportunities
Rowe et al. (45)	Tumor-bearing mice	Formate supplementation enhances CD8+ T-cell fitness and improves anti-PD-1 efficacy	Supporting immune-cell one-carbon metabolism can boost immunotherapy	T-cell support/immunotherapy
Peng et al. (58)	Tumor immune microenvironment	PSPH downregulation potentiates the tumor immune environment and enhances ICB response	Serine biosynthesis is pharmacologically actionable in immunotherapy	Serine-pathway targeting
Ma et al. (79)	Tumor metabolic ecology	Serine enrichment promotes Treg accumulation	Nutrient composition can select suppressive immune populations	Beyond T cells/Treg ecology
Saha et al. (74)	Tumor-cell intrinsic	Serine depletion activates cGAS–STING and promotes antitumor immunity	Serine metabolism has context-dependent effects on tumor immunogenicity	Serine abundance vs depletion
Yuan et al. (116)	Hepatocellular carcinoma	L-methionine enhances CD8+ T-cell killing in HCC	Methionine-directed intervention may support immunity in selected settings	Context-dependent therapy
Wang et al. (111)	Breast cancer	Nuclear PHGDH regulates macrophage polarization	One-carbon-related enzymes also reshape myeloid ecology	Beyond T cells/macrophages
Phelps et al. (45)	Exercise–microbiota–tumor axis	Microbiota-derived formate enhances CD8+ T-cell antitumor immunity and immunotherapy efficacy	Host physiology and microbiota influence one-carbon immunometabolism	Future translational directions

## 5 Epigenetic programming as the bridge between one-carbon metabolism and cancer immunity

### 5.1 One-carbon metabolism supplies methyl groups for immune-state control

One-carbon metabolism occupies a central position in cancer immunity because it directly links nutrient availability to epigenetic regulation. At the core of this connection lies the methionine cycle, which converts methionine into S-adenosylmethionine (SAM), the universal methyl donor used for DNA, RNA, histone, and protein methylation. Through this role, one-carbon metabolism extends far beyond biosynthesis and becomes a regulatory system for cell-state control. In immune cells, especially T lymphocytes, the availability of SAM can influence whether activation programs are sustained, whether exhaustion-associated states emerge, and whether lineage-specific transcriptional identities remain stable (84–86). This principle is particularly important in the tumor microenvironment, where nutrient stress is chronic rather than transient. When methionine becomes limiting, SAM production falls, and the methylation capacity of the cell is correspondingly reduced. Because T-cell activation depends on a coordinated transcriptional program supported by histone and DNA methylation, a reduction in methyl-donor supply can have consequences far beyond metabolism in the narrow sense (87–90). It can alter chromatin accessibility, destabilize effector programs, and bias cells toward dysfunctional states. Thus, one-carbon metabolism functions as a molecular translator that converts extracellular nutrient conditions into durable intracellular changes in gene regulation.

Rather than restating individual methionine-deprivation studies, this section emphasizes the broader epigenetic principle that emerges from them. One-carbon metabolism converts nutrient availability into immune-state stability by controlling the methyl-donor capacity required for chromatin, RNA, and protein methylation. In T cells, insufficient methyl-donor supply may reduce the durability of effector programs and facilitate exhaustion-associated transcriptional states. In tumor cells, altered methylation capacity may influence immune checkpoint expression and innate sensing pathways. Therefore, the central insight is that one-carbon metabolism does not merely affect short-term metabolic fitness; it can imprint longer-lasting immune phenotypes through methylation-dependent regulatory mechanisms.

Conceptually, this makes epigenetic programming the key interface between one-carbon metabolism and cancer immunity. The relevant question is no longer only whether nutrients are sufficient to support cell growth, but whether they are sufficient to maintain the methylation-dependent regulatory architecture required for immune competence. This perspective helps explain why metabolic stress in cancer often produces stable immune dysfunction rather than short-lived metabolic insufficiency. It also suggests that therapies targeting one-carbon metabolism may need to be evaluated not just for their effects on proliferation, but for their consequences on epigenetic fitness in both tumor and immune cells.

### 5.2 Epigenetic control of immune checkpoints and innate sensing

One of the most mechanistically revealing consequences of one-carbon-dependent epigenetic control is its influence on canonical immune escape machinery. If one-carbon metabolism regulates methyl-donor availability and methylation-dependent signaling, then it should be able to shape not only T-cell state but also checkpoint expression and innate immune sensing in tumor cells (91–93). Recent studies strongly support this view. They show that one-carbon metabolism can control the expression of PD-L1, the activity of cGAS, and the responsiveness of the cGAS–STING axis, thereby placing metabolic flux upstream of central immune regulatory pathways. Shang and colleagues provided a key example by demonstrating that the folate cycle enzyme MTHFD2 promotes cancer immune evasion through PD-L1 upregulation. This finding is important because it shifts MTHFD2 from being a purely metabolic enzyme to being a regulator of checkpoint biology. It also shows that tumor one-carbon metabolism does not merely support growth in a background sense; it can actively instruct an immune-evasive phenotype. In this context, PD-L1 expression is not simply a downstream consequence of inflammation or oncogenic signaling, but can also be metabolically sustained through one-carbon-dependent pathways.

Fang and colleagues then extended the concept from checkpoint control to innate immune sensing. They showed that methionine deprivation enhances cGAS activity by preventing its methylation, which is catalyzed by SUV39H1 (94–96). This finding reveals that cGAS itself is subject to one-carbon-dependent epigenetic-like regulation at the protein level, and that nutrient status can tune the threshold for innate immune activation. The importance of this result lies in the fact that it directly connects methyl-donor metabolism to the function of a core DNA sensor that shapes type I interferon signaling and antitumor immunity.

Saha and colleagues provided a complementary perspective by showing that serine depletion activates cGAS–STING signaling in cancer cells and promotes antitumor immunity. This study is especially useful in a review context because it helps integrate serine metabolism, one-carbon flux, and innate immune visibility into a single mechanistic framework. Whereas Shang highlights one-carbon metabolism as a driver of checkpoint upregulation, and Fang shows that methyl-donor limitation can relieve repression of cGAS, Saha shows that perturbing serine availability can similarly enhance innate immune signaling and tumor immunogenicity. Together, these studies support the idea that immune sensing is epigenetically and metabolically tunable.

**Figure 4:**
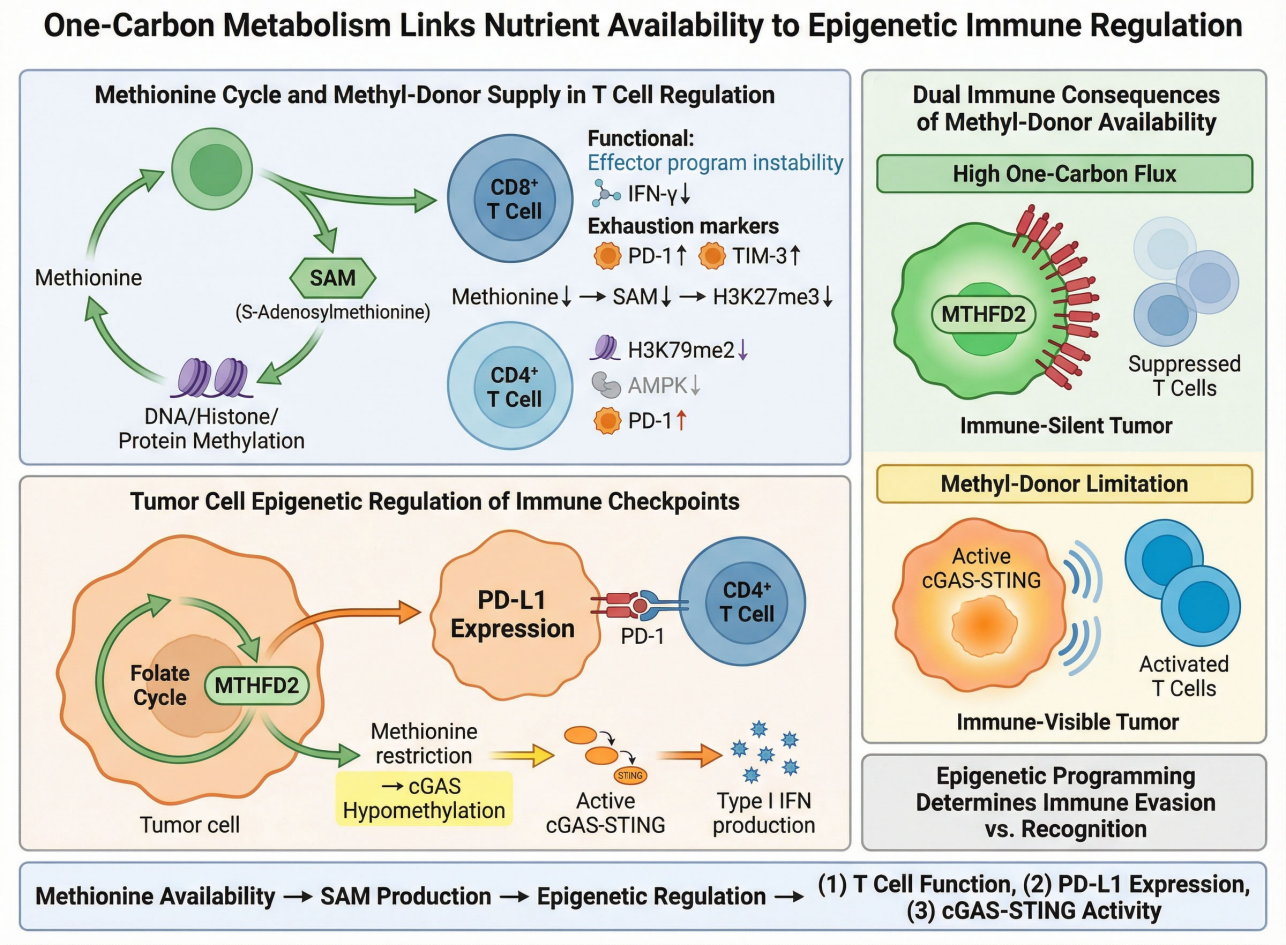
Epigenetic programming bridges one-carbon metabolism and cancer immunity. One-carbon metabolism supplies methyl groups through methionine cycle–derived SAM, thereby regulating DNA, histone, and protein methylation. In T cells, reduced methyl-donor availability destabilizes effector programs and promotes dysfunction or exhaustion. In tumor cells, one-carbon flux influences PD-L1 expression and cGAS–STING activity, indicating that methylation-dependent regulation helps determine whether tumors remain immune-silent or become immune-visible.

These observations also deepen the conceptual significance of one-carbon metabolism in cancer immunity. The pathway is not merely linked to broad features such as proliferation or redox control; it governs highly specific immune escape nodes. By regulating checkpoint expression and innate DNA sensing, one-carbon metabolism can determine whether tumors present themselves as immune-silent or immune-visible. This makes epigenetic programming the most mechanistically dense layer of the one-carbon–immunity interface and arguably the section in which the field’s central logic becomes clearest: metabolism shapes immunity by regulating the molecular machinery through which cells interpret and respond to their environment (97). From a translational perspective, these findings imply that therapeutic intervention in one-carbon metabolism could influence cancer immunity in at least two directions at once: it could reduce checkpoint-mediated suppression and enhance innate immune sensing. However, the same mechanism also raises caution, because one-carbon metabolism supports immune-cell function as well. The challenge moving forward will be to define when and where epigenetic tuning of checkpoint and sensing pathways can be exploited without compromising the methylation-dependent fitness of antitumor lymphocytes. **Figure 4** illustrates how one-carbon metabolism translates nutrient availability into immune regulation through methyl-donor-dependent epigenetic programming. By controlling T-cell functional stability, checkpoint expression, and innate immune sensing, this pathway helps determine whether tumors evade or trigger antitumor immune responses.

## 6 Beyond T Cells: one-carbon metabolism reshapes the broader tumor immune ecosystem

### 6.1 Serine-rich tumors promote Treg accumulation

Discussion of one-carbon metabolism in cancer immunity often centers on effector T cells, especially CD8+ T cells, because of their central role in direct tumor killing. However, the metabolic consequences of one-carbon-related pathways extend well beyond cytotoxic lymphocytes. The composition of nutrients within the tumor microenvironment is selective rather than neutral, and this selective nutrient landscape can favor some immune populations over others. In that sense, metabolic immunosuppression is not only a matter of starving effector cells; it is also a process of ecological selection that enriches suppressive populations.

Ma and colleagues provided a strong example of this principle by showing that serine enrichment in tumors promotes regulatory T-cell accumulation through sphinganine-mediated regulation of c-Fos. This study is particularly important because it reframes serine abundance as an ecosystem-level determinant of immune suppression. Rather than simply supporting tumor cell growth, a serine-rich environment can actively create conditions favorable for Treg expansion or retention (98–101). In this setting, nutrient composition becomes a selective pressure that shapes immune-cell distribution within the tumor. This section therefore shifts the discussion from Treg-intrinsic metabolic stability to tumor ecosystem-level selection. The key point is that serine availability may influence not only how individual immune cells function, but also which immune populations are preferentially maintained within the tumor microenvironment. In serine-rich tumors, immune suppression may arise from positive selection of Treg-dominant niches rather than from effector-cell deprivation alone.

### 6.2 PHGDH and macrophage polarization

The broader immune consequences of one-carbon-related metabolism are not limited to lymphocytes. Increasing evidence indicates that metabolic enzymes associated with serine synthesis and one-carbon pathways can also shape myeloid-cell behavior, including macrophage polarization. This is important because macrophages are major architects of the tumor immune microenvironment, influencing antigen presentation, cytokine balance, angiogenesis, extracellular matrix remodeling, and response to therapy. If one-carbon-related enzymes regulate macrophage fate, then their immunological impact extends well beyond T-cell-centered models.

Wang and colleagues provided a notable example by showing that nuclear PHGDH regulates macrophage polarization through transcriptional repression of GLUD1 and GLS2 in breast cancer (102). This study is particularly interesting because it highlights a noncanonical function of a classical serine synthesis enzyme. PHGDH is traditionally viewed as a metabolic enzyme that supports serine biosynthesis, yet here it also acts in the nucleus as a transcriptional regulator influencing macrophage state. This finding expands the mechanistic vocabulary of the field: metabolic enzymes can function not only through metabolite production but also through direct control of gene expression in immune cells or immune-regulating contexts.

The significance of this result is twofold. First, it demonstrates that one-carbon-related metabolic programs can shape myeloid-cell fate, adding another layer to tumor immune remodeling. Second, it reinforces the idea that the link between metabolism and immunity often operates through gene-regulatory mechanisms rather than through simple substrate supply alone. In the context of this review, the PHGDH study helps prevent an overly narrow focus on CD8+ T cells and underscores that the immune consequences of one-carbon metabolism extend to the broader cellular ecology of the tumor microenvironment (103–108). More broadly, this work suggests that the future of cancer immunometabolism will require integration across immune lineages. A tumor may simultaneously alter methionine availability for T cells, serine abundance for Tregs, and metabolic enzyme activity relevant to macrophage polarization. Such multi-compartment remodeling is likely more representative of real tumors than any single-cell model. Therefore, studies like Wang et al. help move the field toward a more ecosystem-based understanding of metabolic immune regulation.

### 6.3 From immune-cell intrinsic metabolism to metabolic ecosystem remodeling

Taken together, recent work suggests that the most appropriate unit of analysis for one-carbon metabolism in cancer immunity is not an isolated pathway within an isolated cell type, but a metabolic immune ecosystem. One-carbon metabolism acts at multiple levels simultaneously: within tumor cells, where it controls checkpoint expression and immunogenicity; within CD8+ T cells, where it supports effector fitness; within CD4+ T cells, where methionine limitation can promote exhaustion-like states; within Tregs, where serine utilization and redox balance maintain suppressive identity; and within macrophages, where related metabolic enzymes can shape polarization programs. Viewed together, these findings argue that one-carbon metabolism is a distributed regulatory network across the tumor microenvironment rather than a compartmentalized biochemical pathway. This broader ecosystem view is supported by the complementary logic of several key studies. Bian and colleagues showed that tumor cells can deplete methionine and disable CD8+ T-cell epigenetic fitness. Sugiura and colleagues showed that one-carbon metabolism intrinsically governs effector and regulatory T-cell fate. Ma and colleagues demonstrated that serine-rich tumors favor Treg accumulation, while Wang and colleagues showed that a serine-pathway enzyme can help direct macrophage polarization. Each of these studies focuses on a different cellular compartment, yet together they converge on the same idea: nutrient flux and one-carbon-linked regulation help determine not only what immune cells can do, but which immune cells will dominate the tumor microenvironment. This perspective carries important implications for both mechanism and therapy. Mechanistically, it means that one-carbon metabolism cannot be reduced to a single-output pathway such as nucleotide synthesis or methyl-donor generation. Its biological meaning depends on how flux is partitioned across cell types and how each cell type interprets that flux through signaling, chromatin regulation, and fate control. Therapeutically, it means that interventions targeting one-carbon metabolism are likely to have mixed and compartment-specific consequences. The challenge will be to exploit this network in ways that suppress tumor immune escape while preserving, or even enhancing, beneficial immune fitness.

**Figure 5:**
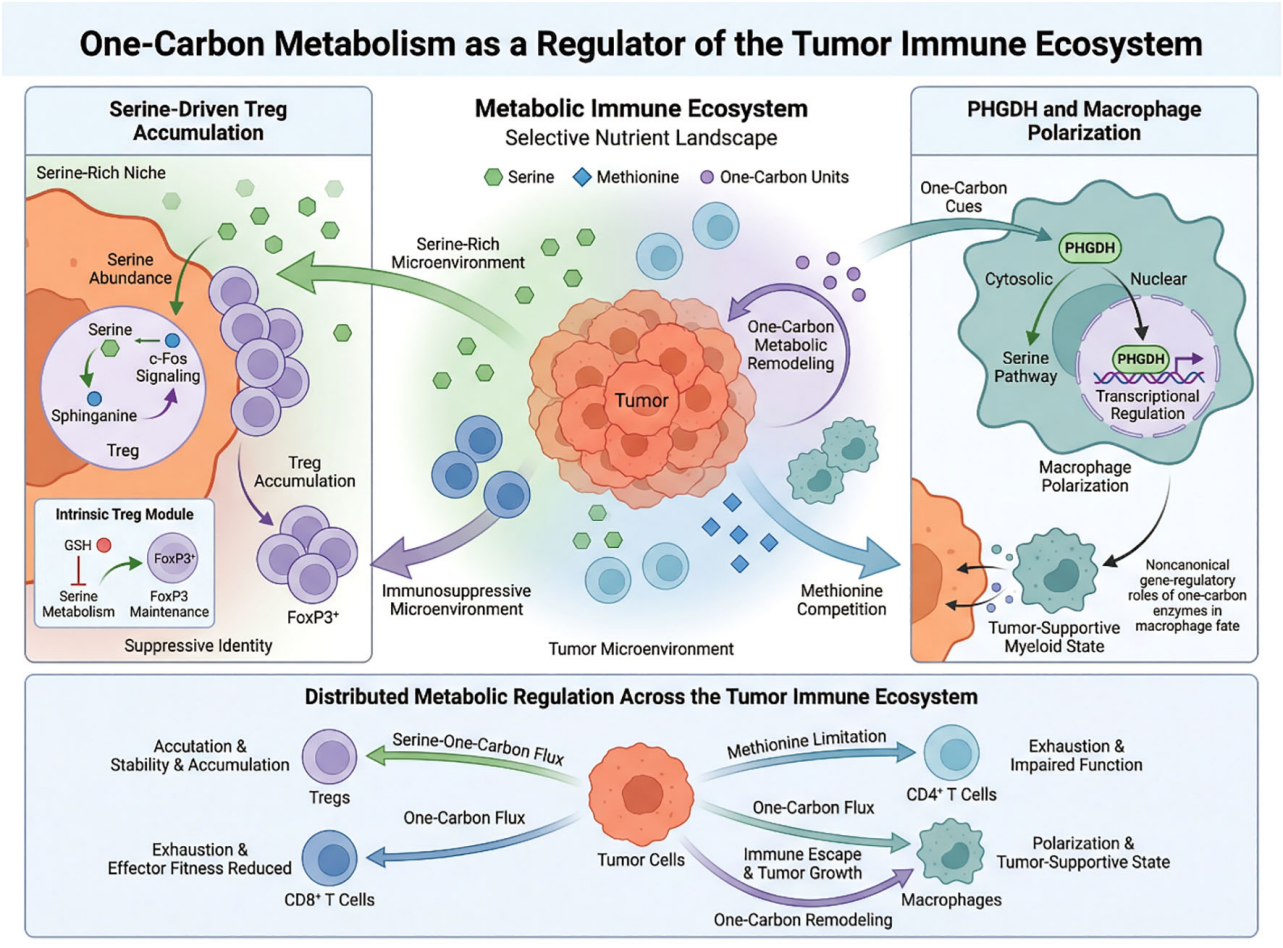
One-carbon metabolism reshapes the broader tumor immune ecosystem. Beyond regulating effector T cells, one-carbon metabolism creates a selective nutrient landscape that favors suppressive immune populations. Serine-rich tumor niches promote FoxP3+ Treg accumulation and stability, whereas one-carbon-related enzymes such as PHGDH influence macrophage polarization. Together, these effects indicate that one-carbon metabolism functions as a distributed regulator of immune-cell composition and functional hierarchy within the tumor microenvironment.

In this light, one-carbon metabolism should be regarded as a framework for understanding how tumors build an immune landscape rather than merely how they fuel growth. The question is not only whether a given metabolite or enzyme supports proliferation, but how it redistributes competitive advantage across tumor cells, effector lymphocytes, suppressive lymphocytes, and myeloid populations. That broader view will likely be essential for designing the next generation of immunometabolic strategies in cancer. **Figure 5** illustrates that one-carbon metabolism acts across multiple immune compartments rather than within isolated cell types. By shaping nutrient availability, Treg ecology, and macrophage polarization, one-carbon metabolism helps construct the broader immune architecture of the tumor microenvironment.

## 7 Therapeutic opportunities: targeting or supporting one-carbon metabolism to improve antitumor immunity

### 7.1 Methionine restriction as an immunomodulatory strategy

Methionine-directed intervention has emerged as one of the most conceptually attractive therapeutic approaches in this field because methionine sits at the intersection of nutrient availability, methyl-donor production, and immune-state regulation (109–114). In principle, restricting methionine could weaken tumor metabolic fitness while simultaneously relieving certain forms of immune suppression. However, the biological consequences of methionine restriction are not uniform. Because methionine is required by both tumor cells and immune cells, its therapeutic manipulation is unlikely to function as a simple on–off switch. Instead, methionine-targeted intervention should be viewed as a context-dependent immunometabolic strategy whose outcome depends on cell type, timing, and the dominant mechanism operating in a given tumor.

Fang and colleagues provided strong support for the idea that methionine restriction can enhance antitumor immunity by altering innate immune sensing. Their study showed that methionine deprivation enhances cGAS activity by blocking its methylation, thereby promoting antitumor immune responses (115). This finding is important because it reveals a mechanism through which limiting methionine does not merely starve tumor cells, but actively increases immune visibility by lifting methylation-dependent repression of innate sensing machinery. In this framework, methionine restriction acts as an immunomodulatory intervention that can reprogram tumor–immune communication, rather than as a purely cytostatic nutrient-limitation strategy. At the same time, methionine targeting cannot be interpreted in a uniformly restrictive direction. Yuan and colleagues reported that L-methionine supplementation can enhance CD8+ T-cell-mediated killing of hepatocellular carcinoma through inhibition of NR1I2/PCSK9 signaling (116). This result serves as a useful counterpoint because it suggests that, under some conditions, increasing methionine availability may support cytotoxic immunity rather than suppress it. Taken together, the Fang and Yuan studies indicate that the therapeutic meaning of methionine depends on which biological process is limiting in a given setting: tumor-intrinsic immune evasion, tumor growth, or immune-cell metabolic competence. For this reason, methionine-targeted intervention is promising but highly context dependent. In some tumors, methionine restriction may preferentially expose tumor-cell vulnerabilities and enhance innate immune activation. In others, excessive methionine limitation may risk weakening T-cell function or compromising immune-cell epigenetic fitness. The translational challenge is therefore not simply whether methionine should be restricted, but under what conditions restriction, supplementation, or selective targeting of methionine handling will best enhance antitumor immunity.

### 7.2 Supporting T-cell one-carbon fitness: formate and related strategies

A second therapeutic direction shifts the emphasis away from restricting tumor metabolism and instead asks whether immune-cell metabolism can be actively supported. This strategy is particularly compelling in the setting of immunotherapy, where reinvigorated T cells may still fail if they cannot overcome metabolic bottlenecks within the tumor microenvironment. From this perspective, one-carbon metabolism is not only a tumor liability to be exploited, but also an immune vulnerability to be rescued (117–119). Formate has emerged as a particularly interesting candidate in this regard because it can serve as a transferable one-carbon unit that supports one-carbon metabolic flux. The translational value of formate-based strategies depends less on the general ability of formate to support one-carbon flux and more on identifying the compartment in which one-carbon insufficiency is functionally limiting. In tumors where CD8+ T cells are present but metabolically constrained, formate supplementation or host-level approaches that increase one-carbon availability may enhance immunotherapy responsiveness. By contrast, in tumors where cancer cells are the dominant formate-responsive compartment, indiscriminate one-carbon support could theoretically reinforce tumor adaptation. Future studies should therefore define biomarkers of immune-cell one-carbon insufficiency, optimal treatment timing relative to checkpoint blockade, and whether systemic formate modulation can be achieved without enhancing tumor metabolic fitness.

Together, these findings support a broader translational concept: improving T-cell one-carbon fitness may enhance the depth and durability of immunotherapy responses. Such approaches may be especially useful in tumors where immune cells are present but metabolically constrained. The challenge will be to identify which patients harbor this type of one-carbon insufficiency and whether formate-based or related support strategies can be deployed safely and selectively in combination with immune checkpoint blockade.

### 7.3 Targeting serine biosynthesis and folate-cycle enzymes

Beyond methionine and formate, several components of the serine synthesis and folate-cycle machinery are emerging as actionable therapeutic nodes (120–124). These targets are attractive because they may regulate tumor growth, tumor immunogenicity, and the immune composition of the microenvironment simultaneously. However, they also raise a central issue in immunometabolism: because many of these enzymes are used by both tumor cells and immune cells, pharmacologic intervention may carry dual and sometimes opposing consequences.

Peng and colleagues showed that downregulation of PSPH, a key enzyme in serine biosynthesis, potentiates a more favorable tumor immune environment and enhances response to immune checkpoint blockade. This result supports the view that *de novo* serine synthesis can help sustain an immune-refractory tumor state and that interrupting this pathway may sensitize tumors to immunotherapy. Importantly, it also suggests that serine-pathway enzymes may serve as both biomarkers and therapeutic targets in tumors with strong one-carbon metabolic dependence. Shang and colleagues, meanwhile, showed that the folate-cycle enzyme MTHFD2 promotes immune evasion through PD-L1 upregulation. This makes MTHFD2 an especially intriguing target because it lies at the interface of tumor metabolism and immune checkpoint control. In principle, inhibiting MTHFD2 could reduce tumor growth and diminish checkpoint-mediated immune escape at the same time. However, Sugiura and colleagues demonstrated that MTHFD2 is also a metabolic checkpoint in effector and regulatory T cells, where it influences purine metabolism and lineage-specific immune function. This dual role immediately complicates therapeutic design. A drug that benefits the immune context by suppressing tumor MTHFD2 could also impair the metabolic fitness of the very immune cells needed for tumor control.

For this reason, *de novo* serine synthesis and folate-cycle enzymes should be regarded as pharmacologically actionable but not biologically simple targets. Their translational value will likely depend on selective delivery, dose optimization, temporal scheduling, or combinations that preserve immune benefit while limiting collateral suppression of T-cell function. In the future, the success of one-carbon-targeted drugs may depend less on whether a target is druggable in principle and more on whether its compartment-specific biology is understood well enough to exploit a therapeutic window.

### 7.4 SAM and methyl-donor-based interventions

Another therapeutic strategy involves manipulating methyl-donor availability directly. Because SAM is the universal methyl donor linking one-carbon metabolism to chromatin state, checkpoint regulation, and innate immune sensing, interventions that alter SAM levels could have broad consequences for tumor progression and immune response (125–129). This approach is appealing because it targets the regulatory output of one-carbon metabolism rather than only its upstream nutrient inputs. At the same time, its breadth means that its effects may be highly dependent on tumor type and biological context.

Mehdi and colleagues reported that SAM can suppress melanoma tumorigenesis and, when combined with immune checkpoint inhibition, enhance anticancer efficacy in both BRAF-mutant and wild-type settings (130). This finding is valuable from a translational standpoint because it suggests that methyl-donor manipulation can cooperate with immunotherapy rather than merely act as an independent metabolic intervention. It also introduces the idea that epigenetic support or reprogramming through SAM may alter tumor susceptibility to immune attack in clinically relevant ways. Yuan and colleagues add another layer to this discussion by showing that methionine supplementation can enhance CD8+ T-cell-mediated killing in hepatocellular carcinoma. Although methionine and SAM are not interchangeable interventions, both studies point toward a broader principle: carefully designed methyl-donor-based strategies may improve antitumor immunity under some conditions. This reinforces the idea that one-carbon-directed therapy cannot be reduced to universal nutrient restriction. In selected settings, supporting methyl-donor availability may actually favor immune competence or increase immunotherapy benefit. These observations suggest that methyl-donor-based interventions deserve further exploration, particularly in combination with checkpoint blockade. However, the field will need to determine whether the key benefit arises from effects on tumor-cell epigenetic programs, on immune-cell fitness, or on both simultaneously. As with other one-carbon-directed therapies, the ultimate value of SAM-based intervention will likely depend on identifying the biological context in which methyl-donor support strengthens antitumor immunity rather than reinforcing tumor adaptation.

### 7.5 Future translational directions: host metabolism, microbiota, and personalized immunometabolic therapy

One of the most exciting directions for the field is the recognition that one-carbon metabolism is shaped not only by tumor-cell pathways and pharmacologic agents, but also by diet, exercise, the microbiota, and broader host systemic state. This broader view matters because many immunometabolic interventions may fail if they are designed only around tumor-intrinsic metabolism while ignoring the systemic sources of nutrient availability and metabolite flux. In the case of one-carbon metabolism, these host-level influences may be particularly relevant because formate, folate-related pathways, and methionine availability are all susceptible to environmental and physiological modulation (131, 132). These observations point toward a broader systems-level model in which one-carbon metabolism is shaped by tumor-intrinsic pathways, immune-cell demand, diet, exercise, microbiota-derived metabolites, and host metabolic state. The translational challenge is therefore to move from generic nutrient modulation toward biomarker-guided immunometabolic therapy. Such an approach would require profiling tumor metabolic dependence, immune-cell one-carbon stress, local nutrient availability, and systemic metabolite supply before selecting restriction-, supplementation-, or microbiota-based strategies.

Taken together, these studies suggest that the future of one-carbon-based cancer therapy may not be limited to direct tumor-targeted drugs. Instead, personalized immunometabolic modulation may incorporate dietary strategies, exercise, microbiota-informed therapy, metabolite supplementation, and rational combinations with immune checkpoint blockade. Such a shift would align one-carbon metabolism with the broader movement toward systems-level precision oncology, in which tumor biology is interpreted in conjunction with host physiology and immune context. **Table 3** summarizes current therapeutic strategies aimed at either targeting tumor one-carbon metabolism or supporting immune-cell one-carbon fitness to improve antitumor immunity.

**Table 3:** Therapeutic strategies targeting or supporting one-carbon metabolism to enhance antitumor immunity.

Therapeutic strategy	Representative target/intervention	Proposed immune effect	Potential advantage	Major caveat	Key references
Methionine restriction	Dietary or metabolic methionine limitation	Enhances innate immune sensing, including cGAS activation	May expose tumor immune vulnerabilities	May also impair immune-cell methyl-donor fitness depending on context	Fang 2023 (115)
Methionine supplementation	L-methionine support	Can enhance CD8+ T-cell killing in selected contexts	May rescue immune-cell metabolic insufficiency	Risk of also supporting tumor metabolism	Yuan 2025 (116)
Formate supplementation	Exogenous formate	Improves CD8+ T-cell one-carbon fitness and anti-PD-1 efficacy	Directly supports immune-cell resilience	Patient selection and optimal dosing remain unclear	Rowe 2023 (45)
Host–microbiota modulation	Exercise/microbiota-derived formate	Enhances systemic one-carbon support for CD8+ T cells	Nontraditional adjunct to immunotherapy	Likely heterogeneous across patients	Phelps 2025 (46)
Serine-pathway inhibition	PSPH or related enzymes	Remodels the TME and enhances checkpoint blockade response	Targets immune-cold metabolic states	Effects may differ by tumor type and immune compartment	Peng 2023 (58)
Folate-cycle targeting	MTHFD2 inhibition	May reduce tumor immune escape and PD-L1 expression	Dual antitumor and immunomodulatory potential	T cells also depend on MTHFD2	Shang 2021 (66); Sugiura 2022 (34)
Methyl-donor modulation	SAM supplementation	May cooperate with ICI and alter epigenetic state	Expands therapy beyond nutrient restriction	Direction of effect may be tumor-context dependent	Mehdi 2023 (130)
Precision immunometabolic combination	Combine one-carbon intervention with ICI	May improve response depth and durability	Fits biomarker-guided therapy design	Requires metabolic stratification and timing optimization	Rowe 2023 (45); Phelps 2025 (46)

## 8 Challenges and open questions

### 8.1 Why are one-carbon interventions context dependent?

A central challenge in this field is that one-carbon interventions often produce context-dependent outcomes. This is not a secondary technical issue but a biological property of the pathway itself. One-carbon metabolism is shared by multiple cell types with different priorities: tumor cells use it to support proliferation, redox adaptation, and immune evasion, whereas immune cells use it to sustain activation, lineage stability, and effector competence. Because the same metabolite or enzyme can serve different functions in different compartments, the direction of therapeutic benefit may vary across tumor types, disease stages, and intervention strategies.

The context dependence of one-carbon interventions can be understood through three variables. First, the affected cellular compartment matters: the same metabolite may support tumor growth in cancer cells but preserve effector function in immune cells. Second, the direction and location of flux redistribution are important: systemic nutrient abundance does not necessarily reflect local nutrient accessibility within the tumor microenvironment. Third, the temporal window of intervention may determine outcome: short-term metabolic perturbation may expose tumor vulnerabilities, whereas prolonged restriction may impair immune-cell fitness. Therefore, one-carbon interventions should not be classified simply as beneficial or harmful. Their therapeutic meaning depends on which compartment is metabolically limited, which pathway branch is being redirected, and whether the intervention is applied during immune priming, effector expansion, checkpoint blockade, or chronic immune exhaustion.

### 8.2 Can we target tumor one-carbon metabolism without impairing immune-cell fitness?

A second major challenge is whether tumor one-carbon metabolism can be therapeutically targeted without harming the immune cells required for tumor control. This issue is particularly clear in the case of MTHFD2. Shang and colleagues showed that tumor MTHFD2 promotes immune evasion through PD-L1 upregulation, making it an attractive antitumor target. However, Sugiura and colleagues demonstrated that MTHFD2 is also a metabolic checkpoint in T cells, where it supports effector and regulatory lineage programs. These studies together create a translational dilemma: the same target that helps tumors evade immunity may also be needed for immune-cell fitness. This dilemma has broader implications for the field. It suggests that dual-use metabolic pathways may not be well served by conventional therapeutic logic in which systemic inhibition is assumed to be beneficial if a target is oncogenic. Instead, the therapeutic window may depend on factors such as dose, timing, tissue distribution, or transient versus sustained inhibition. For example, a short pulse of metabolic inhibition might impair tumor immune evasion while leaving sufficient reserve for immune-cell recovery, whereas continuous exposure could suppress both compartments. Although such strategies remain to be tested, the need for compartment-aware intervention is already evident. A practical consequence is that future one-carbon-targeted therapies may need to be paired with biomarkers that indicate which compartment is most dependent on the pathway at a given time. Without such precision, there is a risk that an apparently rational metabolic therapy could undercut the immune response it is meant to support. Thus, the challenge is not only to identify druggable one-carbon targets, but to define when tumor dependence exceeds immune dependence strongly enough to create a useful therapeutic window.

### 8.3 How should one-carbon metabolism be integrated with immunotherapy design?

The final major open question is how one-carbon metabolism should be incorporated into immunotherapy design in a clinically useful way. The studies reviewed here suggest that integration could take several forms: biomarker-guided patient selection, dietary or nutrient modulation, metabolite supplementation to rescue immune fitness, and microbiota-informed metabolic therapy. However, these approaches will only be effective if they are matched to the dominant metabolic constraint in each tumor–immune setting. Rowe and colleagues support the idea that metabolite supplementation can rescue a specific immune-cell vulnerability and improve PD-1 blockade efficacy. Mehdi and colleagues suggest that methyl-donor-based intervention can cooperate with checkpoint inhibition in melanoma. Phelps and colleagues broaden the concept further by showing that exercise- and microbiota-dependent formate production can enhance immunotherapy responses. Together, these studies indicate that one-carbon metabolism is best thought of not as a standalone therapeutic target, but as a modulatory layer that can be integrated with existing immunotherapies to improve response depth and durability. This implies a future in which immunotherapy design becomes more metabolically informed. Patients may eventually be stratified not only by PD-L1 expression, tumor mutation burden, or immune infiltration, but also by signatures of one-carbon stress, methionine competition, serine-pathway activity, or systemic formate availability. Such an approach could help determine when to restrict nutrients, when to supplement metabolites, and when to combine metabolic intervention with checkpoint blockade. The integration of one-carbon metabolism into immunotherapy design therefore represents both a conceptual shift and a practical opportunity for precision cancer treatment.

## 9 Conclusion

One-carbon metabolism has emerged as a central regulator of cancer immunity rather than a peripheral biosynthetic pathway. Across the studies discussed in this review, a consistent theme is that one-carbon flux influences antitumor immunity at multiple, tightly connected levels: it shapes T-cell fitness through nutrient availability and metabolic checkpoints; it controls epigenetic programming in both tumor and immune cells through methyl-donor supply; it modulates immune escape machinery including PD-L1 and cGAS–STING signaling; and it remodels the broader tumor immune ecosystem beyond CD8+ T cells to include Tregs and macrophages. This body of work also makes clear that one-carbon metabolism offers genuine therapeutic opportunity. Methionine restriction, formate supplementation, serine-pathway targeting, methyl-donor-based intervention, and microbiota-linked modulation all point toward new ways of enhancing antitumor immunity. Yet the same literature also warns against oversimplification. Because one-carbon metabolism is shared across tumor cells and immune cells, successful intervention will require attention to cell-type specificity, nutrient context, and the balance between suppressing tumor adaptation and preserving immune competence. Taken together, the field now supports a broader and more integrated view: one-carbon metabolism is a regulatory framework through which tumors and immune cells compete, adapt, and communicate. Understanding this framework will be essential for the next generation of immunometabolic therapies. The goal moving forward is not simply to inhibit metabolism or to feed immunity, but to reprogram the one-carbon landscape in ways that selectively favor durable antitumor immune control.
